# Targeting the gut microbiota: emerging strategies to enhance healing of diabetic foot ulcers

**DOI:** 10.3389/fendo.2026.1865273

**Published:** 2026-07-22

**Authors:** Zheyan Chen, Wen Wu, Yijie Chen, Feifei Li, Xiaona Xie, Yue Lin, Xueliang Zhang, Qian Ye

**Affiliations:** 1 Zhejiang Chinese Medical University, Hangzhou, China; 2 Department of Plastic and Reconstructive Surgery, Wenzhou Third Clinical Institute Affiliated to Wenzhou Medical University, The Third Affiliated Hospital of Shanghai University, Wenzhou People’s Hospital, Wenzhou, China; 3 Department of Emergency Medicine, Third Clinical Institute Affiliated to Wenzhou Medical University, The Third Affiliated Hospital of Shanghai University, Wenzhou People’s Hospital, Wenzhou, China; 4 Department of General Practice, Third Clinical Institute Affiliated to Wenzhou Medical University, The Third Affiliated Hospital of Shanghai University, Wenzhou People’s Hospital, Wenzhou, China

**Keywords:** diabetic foot ulcer, gut microbiota, gut-immune-skin axis, immune modulation, inflammatory response

## Abstract

Diabetic foot ulcer (DFU) affects up to 34% of diabetic patients, with a 1-year recurrence rate of approximately 40%. This review primarily focuses on type 2 diabetes mellitus (T2DM), the most common form of diabetes associated with DFUs. Emerging evidence shows that gut microbiota critically influences DFUs healing through immune modulation (e.g., Treg/Th17 balance), regulation of inflammatory responses via short−chain fatty acids (SCFAs) that inhibit NF-κB, the gut-immune-skin axis, and systemic effects of microbial metabolites. Microbiota-targeted interventions-probiotics, prebiotics, fecal microbiota transplantation, and dietary strategies-can restore microbial balance and reduce inflammation, thereby promoting DFUs healing. These findings provide a mechanistic foundation for microbiome−based therapies and guide future clinical research.

## 1 Introduction

Diabetic foot ulcers (DFUs) represent a significant clinical challenge and a major complication in patients with diabetes mellitus (1). These ulcers are not only a source of substantial morbidity but also pose a risk of amputation and increased mortality (2), for patients who undergo major amputation, the 5-year mortality rate exceeds 50% (3). The lifetime risk of developing a DFU is estimated to be between 19% and 34%, and approximately 40% of patients will experience a recurrence within one year after initial healing (4). The pathophysiology of DFUs is multifaceted, involving factors such as neuropathy, ischemia, and infection, which contribute to impaired wound healing (5). This review primarily focuses on type 2 diabetes mellitus (T2DM), the most common form of diabetes associated with DFUs. As the prevalence of diabetes continues to rise globally, the burden of DFUs on healthcare systems and the quality of life for affected individuals intensifies. Current treatment approaches—including debridement, offloading, infection control, and revascularization—often yield suboptimal outcomes, with many ulcers failing to heal completely within 12 weeks (6), necessitating the exploration of novel therapeutic strategies that can enhance healing and reduce the incidence of complications associated with DFUs.

Recent research has highlighted the critical role of the gut microbiota in various physiological processes, including metabolism, immune regulation, and inflammation (7). The gut microbiome, a complex ecosystem of microorganisms residing in the gastrointestinal tract, has been shown to influence systemic inflammation and immune responses, both of which are vital in the context of wound healing (8). Dysbiosis, or an imbalance in the gut microbiota, can exacerbate inflammatory conditions and impair healing processes, thereby contributing to the chronicity of DFUs (9). Understanding the interactions between gut microbiota and host physiology may provide insights into new therapeutic avenues for enhancing the healing of DFUs.

Emerging evidence suggests that gut microbiota can modulate the healing of DFUs through several mechanisms. For instance, gut microbiota-derived metabolites, such as short-chain fatty acids (SCFAs), play a significant role in modulating inflammation and promoting tissue repair (10). These metabolites can influence local immune responses and enhance the regeneration of tissues, thereby facilitating wound healing. Furthermore, specific dietary interventions aimed at restoring a healthy gut microbiome have shown promise in improving metabolic control and reducing inflammation, which are crucial for optimal healing outcomes in diabetic patients (11).

This review systematically summarizes the role of gut microbiota in the healing of DFUs, elucidates the underlying mechanisms, and discusses potential clinical applications. By integrating findings from recent studies, we explore how gut microbiota can influence the inflammatory milieu, promote angiogenesis, and enhance tissue regeneration in the context of DFUs. Ultimately, this review aims to provide a comprehensive understanding of the intricate interplay between gut microbiota and diabetic foot ulcer healing, paving the way for innovative treatment modalities that could significantly impact patient care.

## 2 Pathophysiological mechanisms of DFUs

### 2.1 Vascular lesions and ischemia

Diabetes mellitus is a complex metabolic disorder that leads to both microvascular and macrovascular complications, significantly impacting wound healing processes, particularly in the context of DFUs. One of the primary mechanisms by which diabetes affects wound healing is through the development of vascular lesions that result in ischemia. The pathophysiology of DFUs is intricately linked to the presence of peripheral arterial disease (PAD), which is characterized by the narrowing of blood vessels due to atherosclerosis. This vascular impairment leads to reduced blood flow to the extremities, which is critical for delivering oxygen and nutrients necessary for tissue repair and regeneration. Studies have shown that patients with diabetes often exhibit a higher prevalence of both microvascular complications, such as retinopathy and nephropathy, and macrovascular complications, including coronary artery disease and PAD, which together exacerbate the risk of developing DFUs (12, 13).

The ischemic environment created by these vascular lesions intensifies tissue necrosis and prolongs the healing process of foot ulcers. The reduced perfusion not only limits the supply of essential nutrients but also hinders the removal of metabolic waste products, further perpetuating an inflammatory state that is detrimental to wound healing. Research indicates that ischemia can lead to increased levels of reactive oxygen species (ROS), which contribute to oxidative stress and subsequent cellular damage (14). Furthermore, the hypoxic conditions resulting from inadequate blood flow can activate various signaling pathways that impair the migration and proliferation of keratinocytes and fibroblasts, both of which are essential for effective wound healing (15).

Ischemia also exacerbates infection risk by compromising immune responses, allowing bacterial colonization of ulcerated tissue (16). Studies have demonstrated that peripheral arterial disease (PAD) and immune dysfunction are major pathophysiological factors predisposing patients with diabetes to foot ulcers and infections, with infected ulcers often exhibiting more severe ischemic changes than non-infected counterparts (17). This interplay creates a vicious cycle: sustained metabolic dysfunction disrupts fibroblast and immune cell function, perpetuating chronic wounds characterized by persistent inflammation, impaired angiogenesis, and dysfunctional cellular responses, ultimately leading to increased morbidity and, in severe cases, limb amputation (18).To mitigate the effects of ischemia on DFUs, early identification and management of vascular complications are crucial. This includes regular screening for PAD in diabetic patients, as well as timely interventions such as revascularization procedures when indicated. Revascularization can significantly improve blood flow to the affected areas, thereby enhancing healing potential and reducing the risk of complications (19, 20). Additionally, optimizing glycemic control and implementing comprehensive foot care strategies are essential components of managing the risk factors associated with DFUs (21, 22).

### 2.2 Neuropathy and sensory loss

Diabetic neuropathy is one of the most common and debilitating complications of diabetes, affecting approximately 50% of individuals and manifesting as progressive sensory loss in the lower extremities (23). This loss of protective sensation predisposes patients to unrecognized foot injuries, which frequently progress to ulceration, infection, and, in severe cases, lower extremity amputation (24). The relationship between neuropathy and DFUs involves multiple interconnected mechanisms: loss of sensation impairs injury awareness and appropriate response; autonomic dysfunction compromises local blood flow and tissue oxygenation; and neuroinflammation contributes to immune dysregulation that further impedes wound healing (25, 26). The economic burden of diabetic foot disease is substantial. Current diagnostic approaches—including nerve conduction studies, quantitative sensory testing, and emerging screening tools—are essential for identifying at-risk patients, though conventional methods often fail to detect early-stage neuropathy (27). This limitation underscores the need for improved strategies to identify sensory loss before complications develop.

Effective management of diabetic neuropathy requires early detection, patient education, and multidisciplinary care. Lifestyle modifications and emerging therapeutic options targeting neuroinflammatory and metabolic pathways may improve outcomes and reduce DFUs risk (28).

### 2.3 Chronic inflammation and immune dysfunction

Chronic inflammation and immune dysfunction are critical factors that significantly impede the healing process of DFUs. In a hyperglycemic environment, sustained high blood sugar levels lead to a continuous inflammatory response, which is characterized by the persistent activation of immune cells such as macrophages and neutrophils. This activation results in the overproduction of pro-inflammatory cytokines, including tumor necrosis factor-alpha (TNF-α) and interleukins (IL-1β, IL-6), which create a hostile microenvironment detrimental to tissue repair (29, 30). The dysregulation of immune responses manifests as an imbalance between pro-inflammatory and anti-inflammatory signals, further complicating the healing process. For instance, macrophages in DFUs often exhibit impaired polarization, failing to transition from the pro-inflammatory M1 phenotype to the reparative M2 phenotype, which is essential for effective wound healing (31). This dysfunction not only prolongs inflammation but also hinders angiogenesis and the recruitment of necessary cells for tissue regeneration, ultimately leading to chronic non-healing ulcers.

Inflammatory mediators released during this chronic state contribute to tissue destruction and increase infection susceptibility. The resulting systemic inflammation can impair local immune surveillance in the wound, allowing opportunistic pathogens (e.g., *Staphylococcus aureus*, *Pseudomonas aeruginosa*) already present on the skin or introduced from the environment to colonize the wound more easily (29, 32). Subsequent biofilm formation by these bacteria shields pathogens from immune responses and antibiotics, further impairing healing (33). Consequently, the interplay between chronic inflammation and immune dysfunction not only impedes the healing of DFUs but also increases the risk of severe complications such as limb amputation.

Chronic inflammation in DFUs is often associated with systemic complications, including dyslipidemia and kidney dysfunction, prevalent among diabetic patients (34). The systemic inflammatory response can lead to alterations in lipid metabolism, contributing to the development of atherosclerosis and peripheral vascular disease, which further compromises blood flow to the affected areas and delays healing. The complex relationship between local inflammation in DFUs and systemic immune dysregulation underscores the need for an integrated therapeutic approach that addresses both local wound care and systemic metabolic health. Emerging strategies, including immunomodulation therapies and the use of anti-inflammatory agents, hold promise in restoring immune balance and facilitating the healing of DFUs (30, 35). Understanding the intricate mechanisms linking chronic inflammation and immune dysfunction is essential for developing effective interventions aimed at improving healing outcomes for patients suffering from DFUs.

## 3 Composition of gut microbiota and its relationship with diabetes

### 3.1 Basic composition of gut microbiota

The composition of the gut microbiota is a complex and dynamic ecosystem primarily comprising various bacterial phyla, including *Bacteroidetes*, *Firmicutes*, and *Actinobacteria*. Among these, *Bacteroidetes* and *Firmicutes* are the most predominant, contributing significantly to the overall microbial diversity and metabolic functions within the gut. Notably, the *Bacteroidetes* phylum is associated with the breakdown of complex carbohydrates, while *Firmicutes* are known for their role in energy extraction from dietary sources. Additionally, the phylum *Proteobacteria*, although less abundant, includes several pathogenic species that can influence gut health and disease states. The presence of *Actinobacteria*, particularly *Bifidobacterium*, is crucial for maintaining gut health, as these bacteria are involved in fermenting dietary fibers and producing beneficial metabolites such as SCFAs (36, 37).

Diversity and stability of the gut microbiota are essential for intestinal homeostasis and overall health. A diverse microbiome enhances resistance to pathogenic invasions and modulates immune responses. Reduced microbial diversity is linked to obesity, inflammatory bowel diseases, and metabolic disorders (38).The mucin-degrading bacterium *Akkermansia muciniphila* is associated with improved metabolic health, including enhanced insulin sensitivity, reduced adiposity, and decreased systemic inflammation (39).

The gut microbiota is influenced by diet, age, genetics, and environmental exposures. Diets rich in fiber promote beneficial bacteria, enhancing microbial diversity and stability (40). Conversely, diets high in fat and sugar can lead to dysbiosis, characterized by a decrease in microbial diversity and an increase in potentially harmful bacteria. This dysbiosis can contribute to the development of various diseases, highlighting the importance of dietary interventions in modulating gut microbiota composition (41, 42).

### 3.2 The mechanisms linking gut microbiota and diabetes

The gut microbiota of diabetic patients is characterized by reduced diversity and a significant alteration in the composition of microbial populations. Studies have consistently shown that individuals with T2DM exhibit a decrease in beneficial bacteria, such as *Bifidobacterium* and *Faecalibacterium*, while there is often an increase in potentially pathogenic bacteria, including members of the *Enterobacteriaceae* family and other pro-inflammatory taxa (43, 44). At the species level, the butyrate-producing bacterium *Faecalibacterium* prausnitzii and the mucin-degrading *Akkermansia muciniphila* are consistently depleted in T2DM patients, while potentially pathogenic taxa such as Ruminococcus gnavus show increased abundance (45, 46).The chronic inflammatory environment created by dysbiosis not only impairs insulin signaling pathways but also contributes to the progression of diabetes and its complications. Moreover, the reduced diversity of gut microbiota in diabetic patients has been linked to elevated levels of circulating pro-inflammatory cytokines, which can perpetuate a cycle of inflammation and metabolic dysfunction (47).

Conversely, beneficial gut bacteria ferment dietary fibers into short-chain fatty acids (SCFAs)—acetate, propionate, and butyrate—which enhance insulin sensitivity and exert anti-inflammatory effects (48). For example, butyrate is known to strengthen the intestinal barrier, reduce gut permeability, and modulate immune cell function, thereby mitigating the inflammatory responses associated with insulin resistance (49). In diabetic patients, the production of SCFAs is often diminished due to the loss of beneficial bacteria, which may contribute to insulin resistance and impaired glycemic control (45, 50). The gut microbiome not only influences metabolic pathways but also interacts with the immune system, thereby affecting the host’s response to glucose and lipid metabolism. Elucidating the specific mechanisms by which gut microbiota composition influences metabolic health and validating microbiota-targeted interventions in clinical settings remain key priorities for future research (51).

## 4 The mechanism of gut microbiota affecting the healing of DFUs

### 4.1 Immune modulation

The role of gut microbiota in immune modulation is increasingly recognized as a crucial factor in the healing of DFUs. Gut microbiota can influence the functionality of various immune cells, including T cells and macrophages, which are essential for regulating inflammation and promoting tissue repair. These immune cells are pivotal in orchestrating the inflammatory response, which, when dysregulated, can lead to chronic wounds such as DFUs (52). The interaction between gut microbiota and the immune system facilitates a balanced immune response, where pro-inflammatory signals are countered by anti-inflammatory pathways. For instance, specific gut bacteria can promote the differentiation of regulatory T cells (Tregs), which play a vital role in maintaining immune homeostasis and preventing excessive inflammation that can hinder wound healing (53). Furthermore, macrophages, which are key players in the wound healing process, can be influenced by gut-derived signals. Metabolic dysregulation in diabetic wounds leads to accumulation of phenylpyruvate, which promotes M1 macrophage polarization and sustained pro-inflammatory responses via the CD36–PPT1–NLRP3 pathway, thereby impairing tissue repair (54).

### 4.2 Regulation of inflammatory response

The regulation of inflammatory responses is a crucial aspect of the healing process in DFUs, where chronic inflammation significantly impairs wound healing. Recent studies have highlighted the role of microbial metabolites, particularly SCFAs, in modulating inflammatory pathways. SCFAs, which are produced through the fermentation of dietary fibers by gut microbiota, have been shown to inhibit the nuclear factor kappa-light-chain-enhancer of activated B cells (NF-κB) signaling pathway (55). This pathway is a key regulator of inflammation, and its activation leads to the release of various inflammatory mediators that can exacerbate tissue damage and delay healing. By suppressing NF-κB activation, SCFAs can reduce the expression of pro-inflammatory cytokines and chemokines, thereby alleviating the inflammatory milieu that characterizes chronic wounds in diabetic patients. This anti-inflammatory effect is particularly beneficial in the context of DFUs, where prolonged inflammation is a significant barrier to healing.

Chronic inflammation not only hinders the healing process but also contributes to the development of further complications, such as infections and tissue necrosis. By enhancing the resolution of inflammation, SCFAs facilitate the transition from the inflammatory phase of wound healing to the proliferative phase, where new tissue formation occurs. This transition is essential for the successful closure of DFUs, as it allows for the regeneration of skin and underlying tissues, thus restoring the integrity of the affected area (56). Additionally, the interplay between gut microbiota, their metabolites, and the immune system underscores the importance of maintaining a healthy gut microbiome to support wound healing (57).

### 4.3 Promoting angiogenesis and tissue repair

The gut microbiota has been increasingly recognized for its role in promoting angiogenesis, particularly through the modulation of vascular endothelial growth factor (VEGF) expression. which, in the context of wound healing, primarily reflects the activity of the VEGF-A isoform along with contributions from other VEGF family members. VEGF is a critical signaling protein that stimulates the formation of new blood vessels, a process essential for tissue repair and regeneration. Studies have shown that certain gut microbiota can enhance VEGF production, thereby facilitating angiogenesis in various pathological conditions, including DFUs. For instance, SCFAs, which are metabolites produced by gut bacteria during the fermentation of dietary fibers, have been demonstrated to upregulate VEGF expression. This upregulation occurs through the activation of specific signaling pathways that lead to increased endothelial cell proliferation and migration, ultimately contributing to new blood vessel formation. Moreover, the gut microbiota’s influence on systemic inflammation and immune responses can further enhance angiogenic processes, creating a favorable environment for tissue healing. The interplay between gut microbiota and angiogenesis underscores the potential for microbiome-targeted therapies to improve wound healing outcomes in patients with diabetes and other conditions characterized by impaired angiogenesis and tissue repair (58).

**Figure 1:**
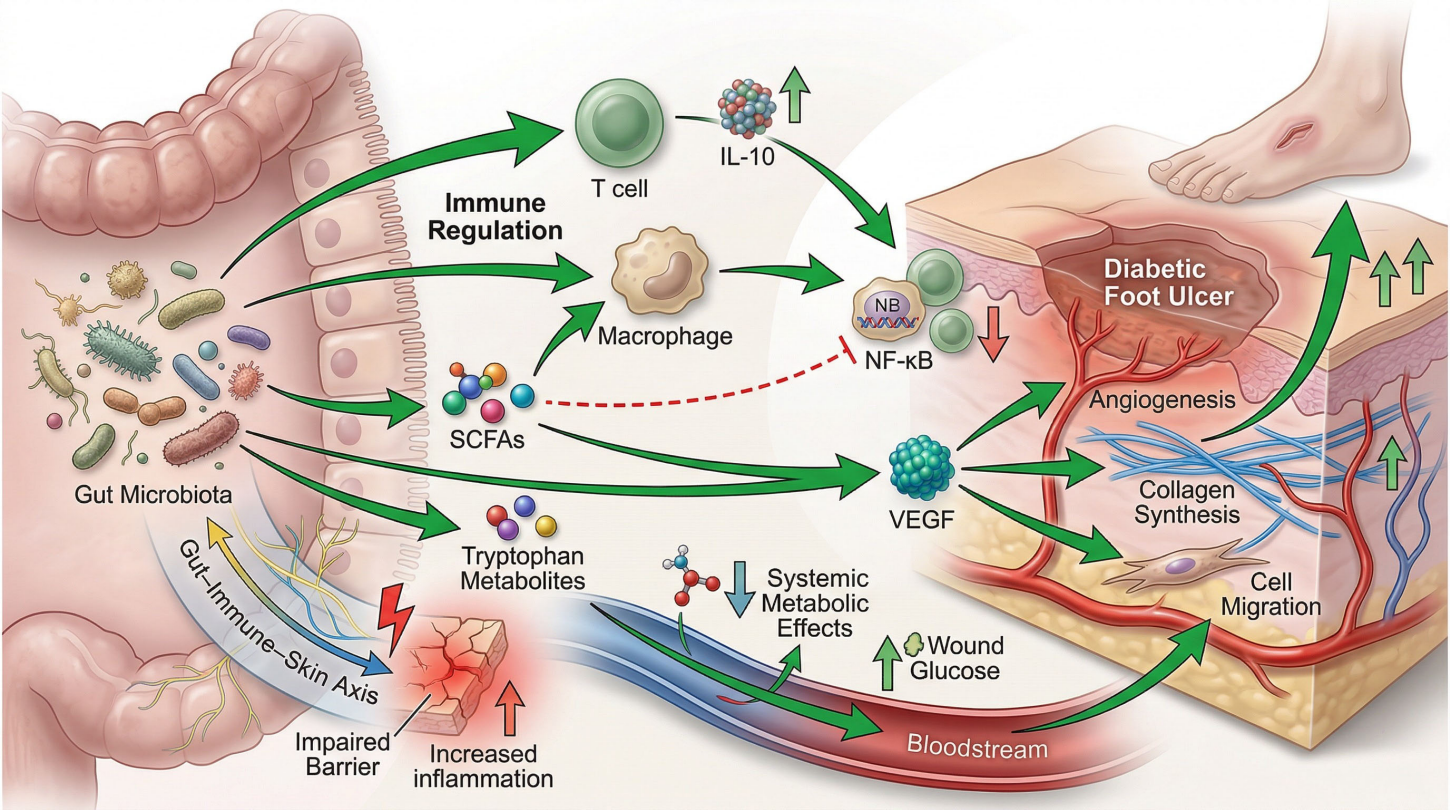
The mechanism of gut microbiota affecting the healing of DFUs. Gut dysbiosis reduces beneficial metabolites (e.g., SCFAs) and increases pro-inflammatory factors (e.g., LPS), compromising intestinal barrier integrity and promoting systemic inflammation. This disrupts wound healing at multiple levels: prolonged inflammation, impaired macrophage polarization, reduced angiogenesis, and delayed re-epithelialization. The gut-skin axis mediates crosstalk between gut microbiota and skin immunity. Microbiota modulation (probiotics, prebiotics, FMT) can restore microbial balance and enhance DFUs healing.

In addition to promoting angiogenesis through VEGF, the gut microbiota also plays a significant role in accelerating wound closure by enhancing collagen synthesis and facilitating cellular migration. For example, certain bacterial species can produce metabolites that stimulate fibroblast activity, leading to increased collagen deposition at wound sites (59). Furthermore, gut-derived SCFAs have been implicated in enhancing the migration of keratinocytes and fibroblasts, which are essential for re-epithelialization and tissue repair. The ability of gut microbiota to promote cellular migration is particularly important in the context of chronic wounds, where delayed healing is often observed. By fostering an environment conducive to cellular movement and collagen production, the gut microbiota can significantly contribute to the acceleration of wound healing processes. This multifaceted role of gut microbiota in promoting angiogenesis and tissue repair highlights its potential as a therapeutic target for enhancing recovery in patients suffering from DFUs and other chronic wounds (60). By harnessing the beneficial effects of gut microbiota through dietary modifications, probiotics, or fecal microbiota transplantation, it may be possible to enhance angiogenic responses and tissue regeneration, thereby addressing the challenges associated with impaired wound healing in diabetic patients and other populations at risk (61). The key mechanisms described in this section are summarized in **Figure 1**.

### 4.4 The role of the gut-immune-skin axis

The gut-skin axis represents a bidirectional communication network linking intestinal microbiota, immune responses, and skin homeostasis. The gut microbiota influences skin health through multiple interconnected mechanisms (62). First, gut microbial metabolites—particularly SCFAs such as acetate, propionate, and butyrate—enter the systemic circulation and exert distal effects on the skin. SCFAs regulate skin immunity through histone deacetylase (HDAC) inhibition and support skin barrier repair via peroxisome proliferator-activated receptor gamma (PPAR-γ) and aryl hydrocarbon receptor (AhR) activation (55); they have been shown to enhance the production of anti-inflammatory cytokines and strengthen the epithelial barrier in the skin, thereby reducing inflammation and promoting healing (63). Second, gut microbiota modulates systemic immune responses by influencing the differentiation and trafficking of immune cells that ultimately migrate to the skin (57). Third, gut dysbiosis can compromise intestinal barrier integrity, leading to increased intestinal permeability and systemic inflammation, which negatively impacts skin barrier function and delays wound healing (64). Dysbiosis has been implicated in various skin disorders, including atopic dermatitis and psoriasis (65). The gut-immune-skin axis is thus a critical pathway through which gut health impacts skin conditions, suggesting that interventions targeting the gut microbiome could provide therapeutic benefits for skin health.

In patients with DFUs, research has indicated significant alterations in both gut and skin microbiota, which collectively exacerbate inflammatory responses and impair healing (56). The skin microbiota plays a direct role in this process: commensal bacteria such as Staphylococcus epidermidis interact with wound-repairing cells to enhance barrier regeneration (66) while pathogenic bacteria including Staphylococcus aureus and Pseudomonas aeruginosa increase in abundance, form biofilms, and perpetuate chronic inflammation and delayed wound healing (56, 67). Meanwhile, a healthy gut microbiome is essential for maintaining immune homeostasis and promoting effective healing responses. Dysbiosis in the gut can lead to the production of pro-inflammatory metabolites that further compromise skin integrity and healing capabilities. Gut microbiota dysregulation can lead to increased metabolic endotoxins, thereby triggering systemic inflammatory responses that are recognized as key pathological mechanisms of DFUs. Notably, the axis operates bidirectionally—skin inflammation can also affect the gut (68, 69). Dermal injury drives a “skin-to-gut axis” that disrupts the intestinal microbiome and intestinal immune homeostasis, with skin wounding changing the composition and behavior of intestinal bacteria and enhancing susceptibility to intestinal inflammation. This bidirectional nature has important clinical implications: in patients with DFUs, the chronic wound not only results from gut dysbiosis but may itself perpetuate gut microbial imbalance, creating a vicious cycle that impairs healing. The recognition of the gut-skin axis as a bidirectional communication system emphasizes the importance of maintaining microbial balance in both the gut and skin to facilitate optimal wound healing. Therapeutic strategies aimed at restoring gut microbiota balance—such as probiotics, prebiotics, dietary modifications, and fecal microbiota transplantation—may simultaneously improve gut health and enhance skin repair, offering a holistic approach to DFUs management (70).

### 4.5 Systemic effects of metabolites

The gut microbiota plays a pivotal role in the production of various metabolites, including SCFAs and tryptophan metabolites, which significantly influence systemic metabolism and immune responses. SCFAs, such as acetate, propionate, and butyrate, are generated through the fermentation of dietary fibers by gut bacteria. These metabolites enter the bloodstream and can affect distant tissues, modulating metabolic processes and immune functions. For instance, SCFAs are known to enhance insulin sensitivity, reduce inflammation, and promote the production of regulatory T cells, which are essential for maintaining immune homeostasis (71). In the context of DFUs, the systemic effects of these metabolites can indirectly facilitate wound healing by improving metabolic abnormalities associated with diabetes. Moreover, tryptophan metabolism, which is influenced by gut microbiota, leads to the production of metabolites such as kynurenine and indole derivatives. These metabolites have been shown to affect immune cell function and may play a role in modulating inflammatory responses, which is crucial in the healing process of DFUs (72).

The interplay between gut-derived metabolites and diabetes-related metabolic dysregulation is particularly noteworthy. Research indicates that SCFAs can alleviate insulin resistance, a common feature in diabetic patients, thereby improving glucose homeostasis (73). This improvement in metabolic control can create a more favorable environment for wound healing, as hyperglycemia is known to impair the healing process through mechanisms such as oxidative stress and inflammation. Additionally, the anti-inflammatory properties of SCFAs contribute to reducing chronic inflammation, which is often exacerbated in diabetic patients and impedes the healing of ulcers (74).

Furthermore, the systemic effects of gut microbiota-derived metabolites extend beyond metabolic regulation to encompass immune modulation. For instance, SCFAs have been shown to enhance the production of anti-inflammatory cytokines while suppressing pro-inflammatory cytokines, thereby promoting a balanced immune response (75). This is particularly relevant in the context of DFUs, where an exaggerated inflammatory response can lead to prolonged wound healing and increased risk of infection. By modulating the immune response, SCFAs and other metabolites derived from gut microbiota can thus play a critical role in facilitating the healing of DFUs. Beyond SCFAs and tryptophan metabolites, other gut microbiota-derived products such as lipopolysaccharides (LPS) and trimethylamine-N-oxide (TMAO) may also contribute to systemic inflammation and metabolic disturbances in diabetic patients. Elevated TMAO levels have been associated with increased diabetes risk and insulin resistance, and recent evidence suggests its potential involvement in diabetic chronic wounds, including DFUs. However, the specific roles of LPS and TMAO in DFU wound healing require further investigation (76, 77).

## 5 Intervention strategies of gut microbiota and their application in DFUs

### 5.1 Application of probiotics and prebiotics

The application of probiotics in the management of DFUs has garnered significant attention due to their potential to improve gut microbiota balance and enhance immune modulation (78). Probiotics, defined as live microorganisms that confer health benefits to the host when administered in adequate amounts, play a crucial role in maintaining homeostasis within the gut environment. In the context of DFUs, the dysregulation of gut microbiota can lead to systemic inflammation and impaired wound healing. By supplementing with probiotics, there is evidence to suggest that these beneficial microbes can restore the balance of the gut microbiome, thus promoting a healthier immune response. Notable examples include multi-strain probiotic formulations combining *Bifidobacterium* and *Lactobacillus* species, synbiotic preparations pairing probiotics with fructo-oligosaccharides (FOS), and prebiotic fibers such as inulin, all of which have shown beneficial effects on glycemic and metabolic parameters in diabetic populations (79–81). Probiotics have been shown to activate the innate immune system, leading to an increase in the production of proinflammatory cytokines that are essential for the wound healing process (82). This immunomodulatory effect is particularly important in DFUs, where the presence of opportunistic pathogens can complicate healing. The administration of probiotics, either orally or topically, can not only enhance the local immune response at the site of the ulcer but also improve overall systemic immunity, thereby reducing the risk of infection and promoting faster healing of the wound (83).

In addition to probiotics, prebiotics also play a vital role in the management of DFUs by promoting the growth of beneficial gut bacteria and improving metabolic and inflammatory states. Prebiotics are non-digestible food components that selectively stimulate the growth and/or activity of beneficial microorganisms in the gut. By providing a substrate for probiotics, prebiotics enhance the efficacy of probiotic supplementation, leading to a more robust and balanced gut microbiome. Synbiotic preparations—combining probiotics with prebiotics such as FOS—have demonstrated superior effects compared to probiotics alone in improving glycemic control and metabolic parameters (84). This synergistic relationship between prebiotics and probiotics can have profound implications for metabolic health, particularly in individuals with diabetes, who often experience altered gut microbiota and increased inflammation. The consumption of prebiotics has been associated with improved glycemic control and reduced inflammatory markers, which are critical factors in the healing of DFUs.

### 5.2 Fecal microbiota transplantation

Fecal microbiota transplantation (FMT) has emerged as a promising therapeutic strategy for restoring a healthy gut microbiome in diabetes and its associated complications, including DFUs (85). Recent studies have demonstrated that FMT can effectively restore microbial balance in diabetic models, leading to improved metabolic outcomes and enhanced wound healing. For instance, a study investigating the effects of FMT in diabetic mice revealed significant improvements in wound healing parameters, including increased closure rates, enhanced re-epithelialization, and greater collagen deposition in skin wounds. This study also identified the IL-17A-mTOR-HIF1α signaling axis as a crucial mediator of the beneficial effects of FMT on wound healing, highlighting the complex interplay between the gut microbiota and host physiology (59). Furthermore, the production of SCFAs by gut bacteria is known to have anti-inflammatory effects, which may further facilitate the healing process in diabetic wounds by modulating local immune responses and promoting angiogenesis (58).

Clinical trials exploring the efficacy of FMT in patients with DFUs are currently underway, aiming to evaluate its potential as an adjunctive therapy for enhancing wound healing in diabetic patients. These trials are critical, as they will provide valuable insights into the safety, feasibility, and effectiveness of FMT in a clinical setting. The underlying mechanisms through which FMT exerts its effects on wound healing are multifaceted and involve not only the restoration of microbial diversity but also the modulation of host immune responses and metabolic pathways (86). For example, the restoration of beneficial bacteria through FMT may enhance the production of metabolites that promote tissue repair and reduce inflammation, both of which are essential for effective wound healing. Additionally, FMT may influence the gut-brain axis and systemic inflammation, further contributing to improved wound healing outcomes.

### 5.3 Dietary regulation and lifestyle interventions

Dietary regulation and lifestyle interventions play a crucial role in managing DFUs by influencing gut microbiota diversity and the production of SCFAs, which are vital for maintaining metabolic health and enhancing wound healing (87). A high-fiber, low-sugar diet has been shown to promote a diverse gut microbiome, which in turn can lead to increased production of SCFAs such as acetate, propionate, and butyrate. These SCFAs have several beneficial effects, including anti-inflammatory properties, modulation of immune responses, and enhancement of epithelial barrier function, all of which are critical in the context of wound healing in diabetic patients. The interaction between dietary components and gut microbiota can significantly alter the metabolic environment, thereby influencing the systemic inflammation that often complicates DFUs. For instance, a diet rich in fiber can foster the growth of beneficial bacteria such as Bifidobacteria and Lactobacilli, which are associated with improved gut health and reduced systemic inflammation. This dietary approach not only supports microbial diversity but also enhances the production of SCFAs that can directly influence the healing process by promoting angiogenesis and collagen synthesis at the wound site (66).

In addition to dietary interventions, physical activity and psychological well-being are essential components of lifestyle modifications that can improve gut-immune function and assist in wound healing (88). Regular exercise has been shown to enhance gut microbiota diversity and promote the production of SCFAs, which can further bolster the immune system’s ability to respond to infections and facilitate tissue repair (89). Exercise increases blood flow and oxygen delivery to tissues, which is particularly beneficial for healing wounds in diabetic patients (90). Moreover, psychological interventions, such as stress management and cognitive-behavioral therapy, can positively affect gut health by reducing stress-induced dysbiosis. Stress is known to alter gut microbiota composition and function, leading to increased inflammation and impaired wound healing. By addressing both physical and psychological aspects of health, patients can achieve a more balanced gut microbiome, which is essential for optimal immune function and effective wound healing.

### 5.4 Combined treatment strategies

The integration of gut microbiota interventions with traditional wound care and anti-infection treatments has emerged as a promising strategy to enhance healing rates in DFUs. Research has shown that diabetic patients often experience dysbiosis, which can exacerbate complications such as infections and delayed healing in DFUs (51). By combining gut microbiota modulation—through the use of probiotics or dietary interventions—with established wound care practices, clinicians may be able to create a synergistic effect that promotes faster and more effective healing. For instance, traditional wound care methods, such as debridement and the application of appropriate dressings, can be complemented by probiotics that restore the balance of gut microbiota, thereby enhancing the immune response and reducing systemic inflammation (91). Studies have indicated that specific gut microbiota profiles can influence the healing process by modulating local and systemic inflammatory responses, thus suggesting a potential pathway through which gut health can be leveraged to improve wound healing outcomes (92).

**Figure 2:**
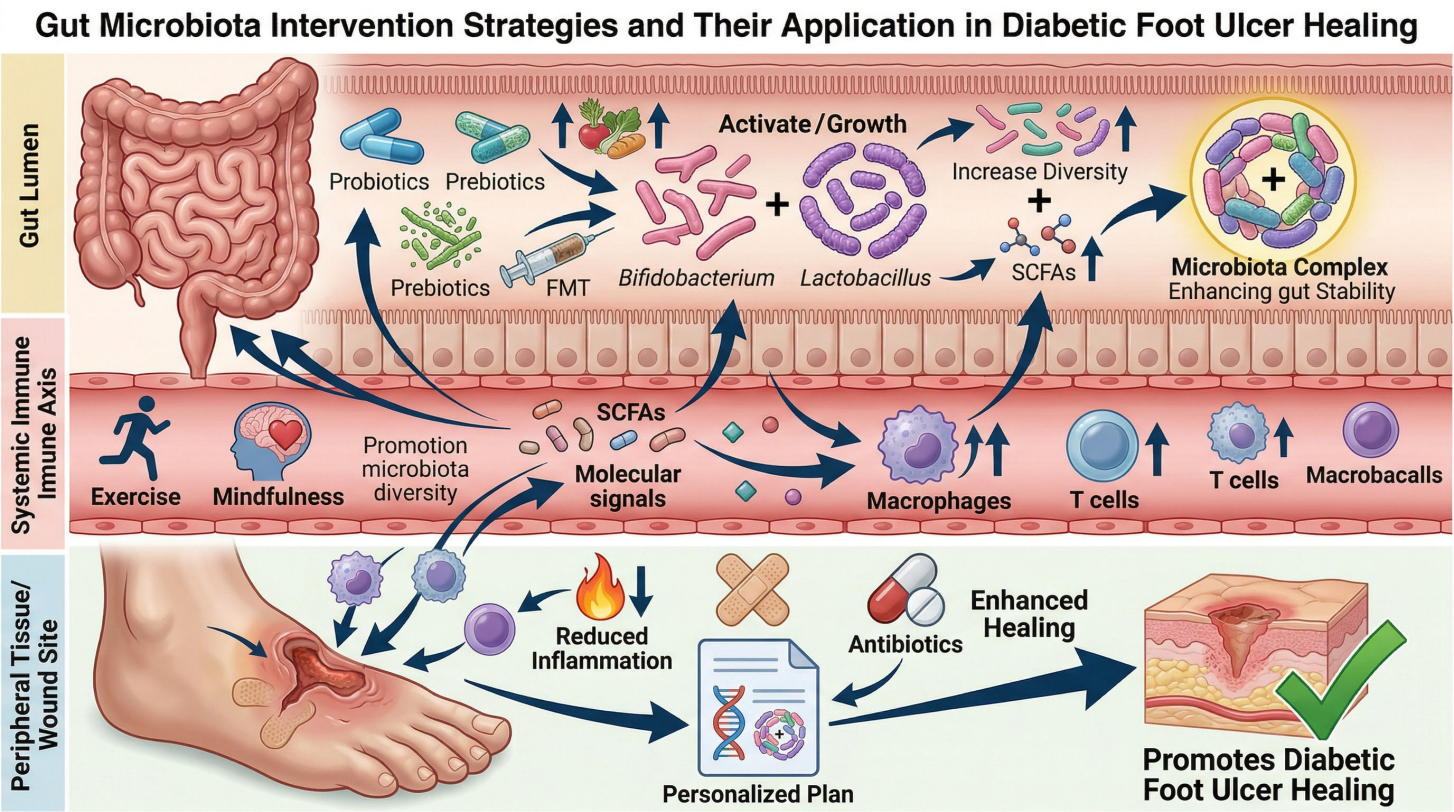
Intervention strategies of gut microbiota and their application in DFUs. This schematic illustrates the pathways through which gut microbiota interventions promote DFU healing. Probiotics (e.g., Bifidobacterium, Lactobacillus) and prebiotics enhance gut microbial diversity and stability, increasing SCFA production. FMT restores microbial balance. These interventions modulate systemic immune responses via molecular signals (SCFAs), regulating macrophage and T cell function, reducing inflammation at the wound site, and ultimately promoting diabetic foot ulcer healing. Lifestyle factors such as exercise and mindfulness further support microbial diversity. Personalized treatment plans integrating these strategies with conventional care optimize clinical outcomes.

Patients with distinct microbiota profiles may respond differently to certain probiotics or dietary modifications, necessitating a tailored approach to treatment. Recent advancements in microbiome research have enabled the identification of specific bacterial strains that may be beneficial for wound healing, paving the way for the development of personalized probiotic therapies that target the unique dysbiosis present in diabetic patients (93). Additionally, integrating gut microbiota assessments into routine clinical practice could allow for more informed decisions regarding the selection of adjunctive therapies, ultimately leading to improved patient outcomes and reduced healthcare costs associated with DFUs. The intervention strategies discussed in this section are illustrated in **Figure 2**.

## 6 Future research directions and challenges

### 6.1 Microbial genomics and multi-omics integration analysis

The integration of multi-omics approaches, particularly metagenomics and metabolomics, has emerged as a powerful strategy to elucidate the functional roles of the gut microbiome and its metabolic products in various health conditions, including DFUs. Metagenomics allows for the comprehensive sequencing and characterization of microbial communities, providing insights into the diversity and abundance of microbial taxa present in the gut. This approach is complemented by metabolomics, which focuses on the analysis of metabolites produced by these microbial communities, thereby revealing their functional capabilities and interactions with the host. Recent studies have demonstrated that specific microbial taxa are associated with the production of metabolites that can influence host metabolism and immune responses, which are crucial in the context of diabetes and wound healing. For instance, SCFAs produced by gut bacteria have been shown to play a significant role in modulating inflammation and enhancing tissue repair processes, which are critical for the healing of DFUs (94). Furthermore, integrating these omics data layers can uncover complex regulatory networks that govern host-microbe interactions, providing a more holistic understanding of the mechanisms underlying diabetic wound healing. Advanced computational methods, such as dynamic Bayesian networks and machine learning algorithms, facilitate the integration of diverse datasets, enabling researchers to identify key microbial signatures and their associated metabolic pathways that correlate with improved healing outcomes (95, 96). However, challenges remain in standardizing these multi-omics approaches and ensuring reproducibility across studies, which is essential for translating findings into clinical applications. The establishment of large, publicly available multi-omics datasets will further enhance our understanding of the gut microbiome’s role in health and disease, paving the way for personalized microbiome-based therapeutic strategies in diabetic wound management (97, 98). As research continues to evolve, the integration of multi-omics data will likely reveal novel biomarkers and therapeutic targets, ultimately leading to more effective interventions for DFUs and other related complications.

### 6.2 Development of personalized microbial intervention strategies

The development of personalized microbial intervention strategies in the context of DFUs is increasingly recognized as a critical aspect of precision medicine. Given the complexity of the human microbiome and its significant role in wound healing, tailoring interventions based on individual gut microbiota profiles is essential. Recent studies have demonstrated that the gut microbiome’s composition can vary significantly among individuals, influencing their responses to dietary interventions and treatments. For instance, research has shown that dietary fiber interventions can lead to variable outcomes in gut microbiota modulation, with some individuals responding positively while others do not (99). This highlights the necessity of developing precise treatment plans that consider the unique gut microbiome characteristics of each patient. By employing advanced sequencing technologies and machine learning algorithms, clinicians can identify specific microbial signatures associated with healing outcomes in DFUs. For example, a study integrating multi-omics approaches found that personalized dietary modifications significantly improved clinical outcomes in pediatric patients with inflammatory bowel disease, suggesting that similar strategies could be applied to DFUs management (100).

Furthermore, evaluating the efficacy of different microbial interventions across diverse patient populations is crucial for establishing effective treatment protocols. The variability in individual microbiomes necessitates a comprehensive understanding of how specific microbial compositions influence healing processes. For example, a recent study indicated that certain bacterial taxa, such as Akkermansia and *Faecalibacterium*, are associated with improved wound healing outcomes in diabetic patients (101). This suggests that interventions targeting these beneficial microbes could enhance healing in DFUs. Additionally, the use of fecal microbiota transplantation (FMT) and probiotics has shown promise in restoring microbial balance and promoting healing in patients with chronic wound (102). However, the success of such interventions may depend on the patient’s baseline microbiome composition, emphasizing the need for personalized approaches.

### 6.3 Development of novel microbial preparations and delivery systems

Innovative drug delivery systems that improve tissue-targeted delivery and modulate the microbiota are gaining traction, aiming to overcome physiological barriers and ensure that probiotics reach the intended sites of action without degradation or inactivation during gastrointestinal transit (103). Moreover, the importance of patient adherence to treatment regimens cannot be overstated. The development of user-friendly delivery systems, such as edible coatings or microencapsulation techniques, can significantly improve the compliance of patients with DFUs. These systems not only enhance the viability of probiotics but also simplify their administration, making it easier for patients to incorporate them into their daily routines. For instance, probiotics delivered in the form of functional foods or beverages can provide a dual benefit of nutritional support while simultaneously delivering therapeutic agents. This approach not only improves adherence but also enhances the overall effectiveness of the treatment by ensuring a consistent intake of beneficial microorganisms that can help restore the microbiota balance and promote wound healing (104).

In addition to improving adherence, the design of these novel microbial preparations must consider the specific microbial profiles associated with DFUs, as certain strains may be more effective in promoting healing and reducing inflammation than others. This personalized approach to probiotic therapy can lead to more targeted treatment strategies, particularly in the context of antibiotic resistance and the need for alternative therapeutic options. Furthermore, combination therapies integrating probiotics with other therapeutic agents, such as antimicrobial peptides or bioactive compounds, may provide synergistic effects that enhance healing outcomes. The use of hydrogels or other biomaterials that release these combinations in a controlled manner at the wound site represents a promising avenue, as such systems can deliver probiotics while providing a scaffold that supports cell growth and tissue regeneration (105).

## 7 Conclusion

The intricate relationship between the gut microbiome and the healing process of DFUs underscores a significant advancement in chronic wound management. As outlined in this review, the gut microbiome plays a pivotal role in modulating immune responses, controlling inflammation, promoting angiogenesis, and mediating the gut-skin axis, collectively contributing to DFUs healing. Gut microbiome interventions—probiotics, prebiotics, and fecal microbiota transplantation—have revealed promising avenues for enhancing DFUs healing by restoring microbial balance and leveraging the beneficial effects of specific microbial populations. The evidence suggests that these interventions could improve clinical outcomes, supporting their integration into standard care practices.

Despite these encouraging findings, several limitations must be acknowledged. First, most mechanistic insights are derived from preclinical animal models, which may not fully recapitulate human DFUs pathophysiology. Second, human studies remain largely cross-sectional, establishing association rather than causation, and the complex interplay between host genetics, diet, medications, and the gut microbiome is often inadequately controlled for. Third, heterogeneity in study designs—including variations in probiotic strains, dosages, treatment durations, and outcome measures—limits direct comparisons and meta-analyses. Future research should focus on elucidating specific pathways, identifying optimal bacterial strains and dosages, and thoroughly evaluating the long-term safety and efficacy of microbiome-based therapies. The variability in individual microbiomes necessitates personalized treatment approaches informed by rigorous clinical validation. Ongoing collaboration among researchers, clinicians, and microbiome specialists will be essential to address the intricate gut-skin interactions impacting DFUs management, paving the way for more effective and sustainable therapeutic strategies.
